# Assessing the Effectiveness of an mHealth Intervention to Support Men Who Have Sex With Men Engaging in Chemsex (Budd): Single-Case and Pre-Post Experimental Design Study

**DOI:** 10.2196/56606

**Published:** 2024-10-04

**Authors:** Corinne Herrijgers, Peter Verboon, Eric Florence, Heidi Vandebosch, Karolien Poels, Tom Platteau

**Affiliations:** 1 Department of Clinical Sciences Institute of Tropical Medicine Antwerpen Belgium; 2 Department of Psychology, Open Universiteit Heerlen Belgium; 3 General Internal Medicine, Infectious Diseases & Tropical Medicine, Antwerp University Hospital Antwerpen Belgium; 4 Department of Communication Studies University of Antwerp Antwerpen Belgium

**Keywords:** mobile health, chemsex, men who have sex with men, MSM, harm reduction, behavioral change, self-efficacy, risk behavior, sexual health, digital intervention, health education, mobile phone

## Abstract

**Background:**

This study focuses on the Budd app, a mobile health intervention designed for gay, bisexual, and other men who have sex with men who participate in chemsex. Chemsex, the use of psychoactive drugs in a sexual context, presents substantial health risks including increased HIV transmission and mental health issues. Addressing these risks requires innovative interventions tailored to the unique needs of this population.

**Objective:**

This study aims to evaluate the effectiveness of the Budd app in promoting drug harm reduction practices among its users, focusing on knowledge, behavioral intention, risk behavior awareness, and self-efficacy.

**Methods:**

The study used a mixed methods approach, combining a single-case experimental design and a pre-post study. A total of 10 participants from an outpatient clinic were recruited, and each attended the clinic 3 times. During the first visit, participants installed a restricted version of the Budd app, which allowed them to report daily mood and risk behavior after chemsex sessions. Phase A (baseline) lasted at least 2 weeks depending on chemsex participation. In the second visit, participants gained full access to the Budd app, initiating phase B (intervention). Phase B lasted at least 6 weeks, depending on chemsex participation, with identical data input as phase A. Participants completed pre- and postintervention surveys assessing behavioral determinants during the first and third visit.

**Results:**

The study observed an increased knowledge about chemsex substances postintervention, with a mean percentage improvement in knowledge scores of 20.59% (SD 13.3%) among participants. Behavioral intention and self-efficacy showed mixed results, with some participants improving while others experienced a decrease. There was also a variable impact on awareness of risk behavior, with half of the participants reporting a decrease postintervention. Despite these mixed results, the app was generally well-received, with participants engaging with the app’s features an average of 50 times during the study.

**Conclusions:**

The Budd app showed effectiveness in enhancing knowledge about chemsex substances among gay, bisexual, and other men who have sex with men. However, its impact on safe dosing behavior, behavioral intention, self-efficacy, and risk behavior awareness was inconsistent. These findings suggest that while educational interventions can increase knowledge, translating this into behavioral change is more complex and may require more participants, a longer follow-up period, and additional strategies and support mechanisms.

## Introduction

### Background

Chemsex, the intentional use of drugs, such as methamphetamine, gamma hydroxybutyrate, gamma-butyrolactone, and mephedrone, before or during sex among gay, bisexual, and other men who have sex with men (gbMSM) [1-3], has emerged as a public health threat due to the potential physical, sexual, and mental health harms [4-6]. Chemsex is characterized by prolonged sexual activity with multiple partners in private settings, facilitated by the use of gay dating apps and the ready availability of recreational drugs in these contexts [1,5].

The combination of illicit drugs, high-risk sexual behavior, digital technology, and psychosocial challenges defines chemsex [1,2]. These factors can interact in ways that exacerbate health risks and lead to multiple negative health outcomes [1,7,8], including increased risk of HIV and sexually transmitted infections (STIs) [9,10], drug dependence and drug overdose [11,12], anxiety and depression [13,14], nonconsensual sex [2,15], and in rare cases even death [16,17]. Therefore, chemsex may contribute to a syndemic among people that engage in this behavior [8,13,18]. Given the potential impact associated with chemsex, there is a need for effective interventions to promote safer participation [19].

The field of mobile health (mHealth) interventions is expanding rapidly, and emerging technologies hold promise for improving public health by providing cost-effective interventions to a wide audience. A recent systematic review shows that mHealth interventions can offer private, anonymous, tailored, and convenient access to behavior change interventions that are acceptable for gbMSM across sociodemographic groups [20]. This complements traditional health care consultations that are often complicated by fears of stigma and legal issues related to chemsex activities, affecting both health care providers and their clients [21-23]. mHealth tools provide a confidential, less stigmatizing alternative for these traditional services, thereby optimizing the access to relevant information. mHealth apps, in particular, have been increasingly shown to impact health awareness and behavior change, such as smoking cessation [24], sexual health improvement [25], mental health promotion [26,27], reduced alcohol consumption [28], and promotion of physical activity [29].

Ideally, mHealth interventions that focus on gbMSM participating in chemsex should take into account the complex interactions between drug use, sexual behavior with increased risk of HIV and STI infection, and associated health problems as these can have multiplicative effects and potentially reduce the overall burden associated with chemsex. Therefore, developing a mobile app for individuals engaging in chemsex may be a promising approach to promote safer participation in these activities [30].

A recent randomized controlled trial conducted in Hong Kong found that a brief web-based intervention using a harm reduction approach was effective to enhance self-efficacy in refusing risky sexual behavior and chemsex, and improve the uptake of HIV testing among gbMSM engaging in chemsex [31]. Results also confirmed initial evidence that an mHealth intervention can reduce both gbMSM’s intention and their actual engagement in chemsex. However, there is still a need for additional research to develop and evaluate web-based interventions that specifically address the needs of gbMSM who engage in chemsex [32].

Considering the complex interplay of substance use and high-risk sexual behavior associated with chemsex, mHealth interventions targeting gbMSM in this context should address these multifaceted health challenges comprehensively. Several existing mHealth interventions focus primarily on sexual risk reduction among gbMSM engaging in chemsex [20]. To address this complex interplay, we developed an mHealth app “Budd,” using the intervention mapping protocol (IMP) for a systematic and evidence-based approach [33]. A description of Budd’s development process is published elsewhere [7].

Budd aims to address multiple objectives, including the application of drug harm reduction measures, conscious planning of chemsex participation, access to support services, adhere to HIV medication or preexposure prophylaxis (PrEP) for HIV, and to assist others during chemsex events in case something goes wrong. These program objectives are derived from results from a previous study on specific needs among gbMSM who engage in chemsex [7]. This study primarily evaluates the app’s effectiveness in achieving its first goal, that is, to assess harm reduction measures that are taken during chemsex. We selected this program objective based on the results from the needs assessment [7]. Many components of the app are geared toward this goal. For example, the app includes drug information, a drug combination tool, articles about drug-related topics, emergency information, a notebook with timestamps, etc.

### Rationale

Despite a growing body of evidence that affirms the effectiveness of mHealth interventions in improving sexual health among gbMSM [34-36], there is still a significant gap in knowledge when it comes to the effectiveness of mHealth interventions in preventing and reducing the harms associated with chemsex specifically. To the best of our knowledge, the above described study conducted in Hong Kong is unique in its kind [31].

Our Budd app was developed using a comprehensive approach to chemsex, aiming to address a spectrum of 5 programmatic goals. However, for this manuscript, we narrow our focus and examine specifically the app’s efficacy to put drug harm reduction measures into practice. In this light, our study aims to investigate whether the Budd app can positively influence key behavioral determinants related to harm reduction in the context of chemsex. These determinants include knowledge, behavioral intention, risk behavior awareness, and self-efficacy. Our second aim is to assess whether this tailored focus leads to an observable positive change in the practice of safe dosing of substances during chemsex sessions.

The findings of this research could guide the development of future interventions to address the unique needs of this population and ultimately minimize the adverse health outcomes associated with chemsex.

## Methods

### Recruitment

The study was carried out at the outpatient clinic of the Institute of Tropical Medicine (ITM) in Antwerp (Belgium). ITM’s outpatient clinic consists of an HIV-treatment center, a low-threshold HIV and STI testing center and a PrEP-consultation. A total of 12 participants were recruited. A total of 9 (75%) participants were recruited through PrEP or HIV and STI consultation at ITM. The remaining 3 (25%) were recruited from respondents who had participated in interviews during an earlier stage of Budd’s development process and agreed to take part in further research projects. These initial interviews were focused on understanding harm reduction practices and assessing the needs of gbMSM who engage in chemsex [32]. The inclusion criteria of participants are shown in Textbox 1.

**Participant inclusion criteria.**

**Inclusion criteria**

- Aged at least 18 years
- Self-identifying as a male member of the lesbian, gay, bisexual, transgender, and queer+ community
- Being able to understand and express oneself in Dutch
- Having intentionally used drugs (crystal methamphetamine, mephedrone, gamma hydroxybutyrate, gamma-butyrolactone, 3-4 methylenedioxymethamphetamine, ecstasy, cocaine, ketamine, or new psychoactive substances) before or during sex within the past 3 months
- Owning a smartphone

Of the 12 participants, 2 (17%) were excluded from the analysis; one decided to withdraw during the study, and another stopped filling out the daily self-reports. This left a final analysis group of 10 participants, of which only 1 (10%) individual was from the group that had previously participated in the early-stage interviews.

### Ethical Considerations

This study was reviewed and approved by the institutional review board of ITM, on September 8, 2021 (ref 1520/21). The approval ensured that the study adhered to ethical guidelines for research involving human participants. Informed consent was obtained from all participants involved in the study.

During their first visit to the clinic, participants were provided with comprehensive information about the study’s purpose, procedures, potential risks, and benefits.

The study adhered to strict privacy and confidentiality measures. Data from the Budd app were stored on Combell, a hosting service compliant with ISO 27001:2013 standards, ensuring high information security. A processing agreement with the app developer outlined data handling and archiving. Participant accounts were linked to unique study codes, with no personal identifiers stored in the app. Names and emails were kept in a separate file, accessible only to authorized staff, ensuring pseudonymization. Google Analytics was used for app use tracking with participant consent. Pre- and poststudy surveys were conducted via KoBoToolbox, installed on ITM servers with advanced security measures such as firewalls and password protection. Only the project manager had access to these data. The study complied with the General Data Protection Regulation, with data fully anonymized 1 year after collection and retained for 5 years. These protocols ensured participant confidentiality and data security throughout the study.

Participants were compensated for their time and effort. A total fee of €100 (US $111.55) was provided to each participant for completing the entire research process. This compensation was structured as follows: €30 (US $33.47) for the first visit, €30 (US $33.47) for the visit between phase A and phase B, and €40 (US $44.62) for the final visit at the end of the study.

### Study Design

The effectiveness of the Budd app was evaluated using a combination of a pre-post study design and a single-case experimental design (SCED) with multiple baselines. SCED is a research methodology that is increasingly used to evaluate the preliminary effectiveness of health interventions [37,38].

In the SCED method, the dependent variables are assessed repeatedly for each participant across different phases, with a baseline (A) and an intervention phase (B), where each participant serves as his or her own control [39]. This methodology was applied to the assessment of mood and self-reported safe dosing practices. Mood was assessed daily, and self-reported safe dosing practices were recorded after participants had attended a chemsex session. However, due to the exploratory nature of this intervention and constraints in time and resources, the frequency of data collection for self-reported safe dosing practices occasionally fell below the recommended 5 data points per phase as outlined in SCED guidelines. We acknowledge this limitation, which suggests that the study’s findings should be viewed as preliminary.

Behavioral determinants such as knowledge, behavioral intention, awareness of risk behavior, and self-efficacy were measured using a pre-post study design. These determinants were assessed before the intervention at the first clinic visit and after it concluded at the third clinic visit.

Our study used a nonconcurrent SCED, with participants beginning the intervention at various random start points (Multimedia Appendix 1). This design was selected to manage logistical constraints while ensuring the integrity of the experimental conditions, as recommended by Single-Case Reporting Guideline in Behavioral Interventions 2016 guidelines [40].

**Figure 1:**
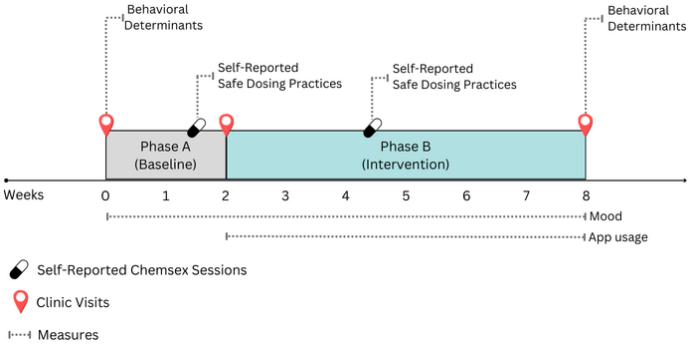
Study design for evaluating the Budd app among gay, bisexual, and other men who have sex with men who participate in chemsex.

This variation helps to assess the impact of the intervention more accurately and determine whether changes observed can be attributed to the intervention [41]. It is important to note that chemsex sessions were not scheduled as part of the study protocol. Instead, these were natural occurrences that participants self-reported during the study period. Figure 1 shows an overview of our study design.

### Procedure

Each participant was required to attend the clinic 3 times. During their first visit, the study’s procedures were explained, and informed consent was provided. Participants completed a demographic questionnaire and a baseline survey on the behavioral determinants of chemsex behavior. In addition, they installed a restricted version of the Budd app on their smartphones, which only allowed them to report their daily mood and risk behavior after each chemsex session but did not (yet) grant them access to the intervention’s content. For the transition from phase A to phase B, participants visited the clinic a second time to get full access to the content of the Budd app. During this visit, the researcher assessed whether participants required any additional assistance or support.

Phase A started after this baseline assessment and was initially set to last at least 2 weeks. However, this phase was extended as needed if a participant did not engage in a chemsex session within the initial 2-week period, ensuring that each participant had at least 1 session to report before moving to phase B. Phase B (intervention) started after the completion of phase A and was intended to last 6 weeks. Similar to phase A, the duration of phase B was flexible and extended if participants did not report any chemsex activity during the intended period. This phase only concluded once the participant had attended and reported at least 1 chemsex session.

During a third and final visit to the clinic, after having finalized phase B, participants completed the poststudy assessment, where they filled in the same behavioral determinants questionnaire as during the prestudy assessment. Importantly, at the initial intake, participants were clearly informed that there was no expectation or pressure from the research team to engage in chemsex. The design of both phases was intended to capture natural behaviors under the influence of the intervention, without promoting chemsex activity.

### Intervention

The Budd app is an mHealth intervention to support and inform gbMSM who participate in chemsex, reduce the negative impacts associated with chemsex, and encourage more mindful participation. It consists of 2 main components:

Information module, covering the following:

- Articles on chemsex drugs, harm reduction, and safer sex
- Personal testimonials from chemsex participants (“Chemsex Stories”)
- Specific information, including effects and dosage, on commonly used substances
- A combination tool, which is a tool to check interactions between up to 4 different substances, with risk indicators
- Local health care and support resources, where an overview of local health care professionals, counseling services, and testing locations are provided
- Emergency info, where concrete step-by-step guidance on handling emergency situations such as overdose is provided

Individual support and planning, including the following:

- Events page and preparation tool, which allows users to plan and prepare their chemsex sessions using an 8-item planning questionnaire
- Check-in and out feature, a button that enables users to track progress, mood, and monitor the time between drug doses
- Reflection and stats, a “personal stats” page that offers various data, including mood tracking and behavioral details on each session; this enables users to reflect on their experience and compare pre- and postsession intentions

In summary, the Budd app offers a multifaceted approach to chemsex participation, providing users essential information, individualized planning tools, and resources to engage in chemsex in a more conscious and safe manner. A comprehensive overview of the development and content of the app is provided in a dedicated article [7].

### Data Collection

Data were collected between October 2021 and April 2022. The primary goal of the study is to assess the effectiveness of the Budd app in helping chemsex users to apply substance use harm reduction measures, our first program objective.

### Primary Outcome Measures

#### Behavioral Determinants

The primary outcome measure is the change in behavioral determinants. The IMP [42], which guided the development of our app [7], suggests that changes in determinants of behavior may serve as a necessary precursor to changes in actual behavior [43,44]. Subsequently, we expect changes in the behavioral determinants rather than changes in participants’ actual behavior after using the app for a limited time (6 weeks). The specific behavioral determinants examined in this study include knowledge, behavioral intention, awareness of risk behavior, and self-efficacy. We focus on these determinants as they are primarily influenced by the 3 core behavior-change methodologies that we use in the Budd app: providing information, self-monitoring of behavior, and goal-setting [7].

#### Knowledge

To measure the behavioral determinant “knowledge,” we developed a 34-item multiple-choice questionnaire consisting of questions that focus on substances (properties, dosage, effects, and risks) related to chemsex (Multimedia Appendix 2), with 4 answering categories for each question. All answers to the 34 questions are to be found in the Budd app. The scores ranged between 0 and 34, with a higher score representing more knowledge. The questionnaire was reviewed and validated by an independent medical specialist in substance use, who was not linked to the study. We compared participants’ scores on the knowledge questionnaire before and after participation in the study to assess improvement in knowledge on chemsex-related substance use.

#### Behavioral Intention, Awareness of Risk Behavior, and Self-Efficacy

To assess the determinants “Behavioral intention,” “Awareness of risk behavior,” and “Self-efficacy,” we built on our change objectives formulated during step 2 of the IMP [7]. Each change objective related to the behavioral goal of “applying drug use harm reduction measures” was transformed into a statement. The exact formulation of each statement was developed following the principles outlined in the theory of behavioral change for constructing scales of behavioral determinants [45]. Participants rated each statement on a Likert scale from 1 (“strongly disagree”) to 5 (“strongly agree”). The statements were selected depending on their relevance for the Budd app. A detailed overview of the determinants included in the study and the questionnaire can be found in Multimedia Appendix 3.

“Behavioral intention” was assessed by participants as a score computed with their answers on the matching statements. The statements were designed to measure participants’ intentions regarding safe participation in chemsex, which included their intention to avoid dangerous drug combinations, dose correctly, wait sufficiently before redosing, and bring essential items for harm reduction to a chemsex party (eg, snuff tube, needles [if applicable], and measuring tube).

A similar approach was used to evaluate “Awareness of risk behavior.” Relevant statements contained participants’ awareness of the types and doses of chems they are taking during a chemsex party, and their awareness of the timing of their chem use.

The last behavioral determinant, “Self-efficacy,” was assessed in the same way. Statements to evaluate “Self-efficacy” probed for participants’ confidence to engage in safer chemsex behaviors, such as avoiding dangerous drug combinations, dosing correctly during a chemsex party, waiting an adequate amount of time before redosing, and bringing harm reduction materials to a chemsex date or party.

### Secondary Outcome Measures

#### Sociodemographic Variables

We collected sociodemographic data from participants, aiming to characterize the study population. These data include the participant’s age, highest level of education, and current employment status. In addition to sociodemographic data, we also assessed participants’ engagement in chemsex before participation in the study. Specifically, we inquired whether participants have engaged in chemsex in the past 2 months and, if they have, how often they participate in chemsex on average. We further gathered information on the types of substances (“chems”) that participants have used during chemsex. This questionnaire assessing sociodemographic variables can be found in Multimedia Appendix 4.

#### Self-Reported Safe Dosing Practices

Unlike the assessment of the primary outcome measures (behavioral determinants, see above) that were assessed before and after the access to the Budd app, this chemsex-related risk behavior was provided after each attended chemsex event. This self-reported risk behavior was evaluated using a 14-item risk behavior questionnaire. The content of this risk behavior questionnaire was based on a literature review, feedback from the advisory group of stakeholders and interviews with potential users during earlier stages of intervention development [7,32]. The questionnaire consisted of questions related to drug use, chemsex session duration, STI transmission prevention, negative experiences during and after chemsex, peer pressure, and reciprocal consent during chemsex. Descriptive results of these findings have been published in a separate article [46].

Although more information was collected, for this analysis, we focus on self-reported safe dosing practices. Safe dosing practices were measured using the statement “I dosed as safely as possible,” which participants rated using a Likert scale ranging from 1 (strongly disagree) to 5 (strongly agree).

#### System Use Data

We collected use data through Google Analytics. We aggregated total time spent on the app, number of pages viewed, number of app sessions, mean pages per session, and mean session duration. Technology-delivered interventions have the potential to improve users’ behaviors and outcomes; however, this is contingent on user engagement with the intervention [47]. Use data can provide valuable insights into how users interact with the intervention, which can inform our understanding of engagement.

#### Mood

Mood was measured daily in our study through the app’s built-in “mood journal” using a single-item scale: “How are you doing?.” The possible answers were excellent, very good, good, fair, and poor [48]. For analytical purposes, we reversed the scoring such that a higher score indicates a better mood.

Mood can impact a person’s willingness to take risks, which is crucial in the context of health-related decision-making and adherence to harm reduction strategies. The affect infusion model argues that being in a good mood can lead people to take more risks [49]. This happens because they see more of the potential rewards and less of the potential dangers of risky actions. In contrast, the mood-maintenance hypothesis states that people in a good mood might avoid taking risks to keep feeling good [50]. Similarly, the mood repair hypothesis states that people in a bad mood might take more risks as a way to try and feel better [51]. Empirical studies on mood and risk-taking present mixed outcomes. Some research supports the idea that a positive mood leads to more risk-taking and a negative mood leads to less [52-54]. However, other studies have found the opposite effect [55,56].

Understanding its fluctuations over time may provide valuable insights into the interplay between mood, behavior, and app use.

### Data Analysis

To assess the effectiveness of the Budd app in promoting harm reduction practices among gbMSM engaging in chemsex, we used a combination of visual and statistical analysis techniques.

#### Visual Analysis

Following the principles of SCED, we interpreted our data through visual analysis [37]. To facilitate this, both mood and safe dosing behavior were depicted using time series graphs. The y-axis represented the measured outcomes (safe dosing behavior and mood), while the x-axis corresponded to the time line of participated chemsex events and measured days, respectively. Phase A (baseline) and phase B (intervention) were distinguished using color-coded lines; blue for phase A and red for phase B. For the safe dosing behavior graph, a vertical dashed line indicated the transition between phases, providing a clear visual separation of the intervention’s onset. The analyst was not blinded to the conditions during data analysis.

#### Statistical Analysis

We explored individual changes in behavioral determinants by comparing the means of pre- and postintervention scores. This analysis allowed us to quantitatively assess the Budd app’s impact on participants’ knowledge, behavioral intention, risk behavior awareness, and self-efficacy. Sociodemographic variables and system use data were described descriptively to characterize the study population and their engagement with the app.

## Results

### Sociodemographic Information

Table 1 shows the characteristics of the participants at the baseline assessment.

**Table 1:** Baseline characteristics of participants in the Budd intervention effectiveness study.

Participant	Age (y)	Level of education	Professional status
1	28	Professional bachelor’s degree	Full-time
2	54	Secondary diploma	Full-time
3	51	Secondary diploma	Full-time
4	58	Master’s degree	Unemployed
5	40	Professional bachelor’s degree	Full-time
6	31	No secondary diploma	Full-time
7	37	Master’s degree	Full-time
8	34	Secondary diploma	Full-time
9	33	No secondary diploma	Full-time
10	26	Master’s degree	Full-time

### Chemsex Participation

Table 2 presents the frequency of chemsex participation among the participants before and during the intervention. For each participant, the number of chemsex sessions during phase A (when the app was not in use) is compared with the number of chemsex sessions during phase B (when the app was in use). The table also includes the total number of chemsex sessions for each participant across both phases, as well as the total hours spent in chemsex sessions.

**Table 2:** Overview of chemsex session participation before and during the Budd intervention.

Participant	Chemsex sessions phase A	Duration of phase A (days)	Chemsex sessions phase B	Duration of phase B (days)	Total sessions	Total hours at chemsex sessions
1	4	19	6	63	10	390
2	3	20	4	59	7	105
3	1	18	3	64	4	11
4	2	19	2	72	4	43
5	7	27	4	42	11	88
6	5	44	3	46	8	128
7	1	33	1	51	2	28
8	1	31	8	47	9	187
9	1	31	1	47	2	47
10	1	15	3	46	4	52

### Mood

**Figure 2:**
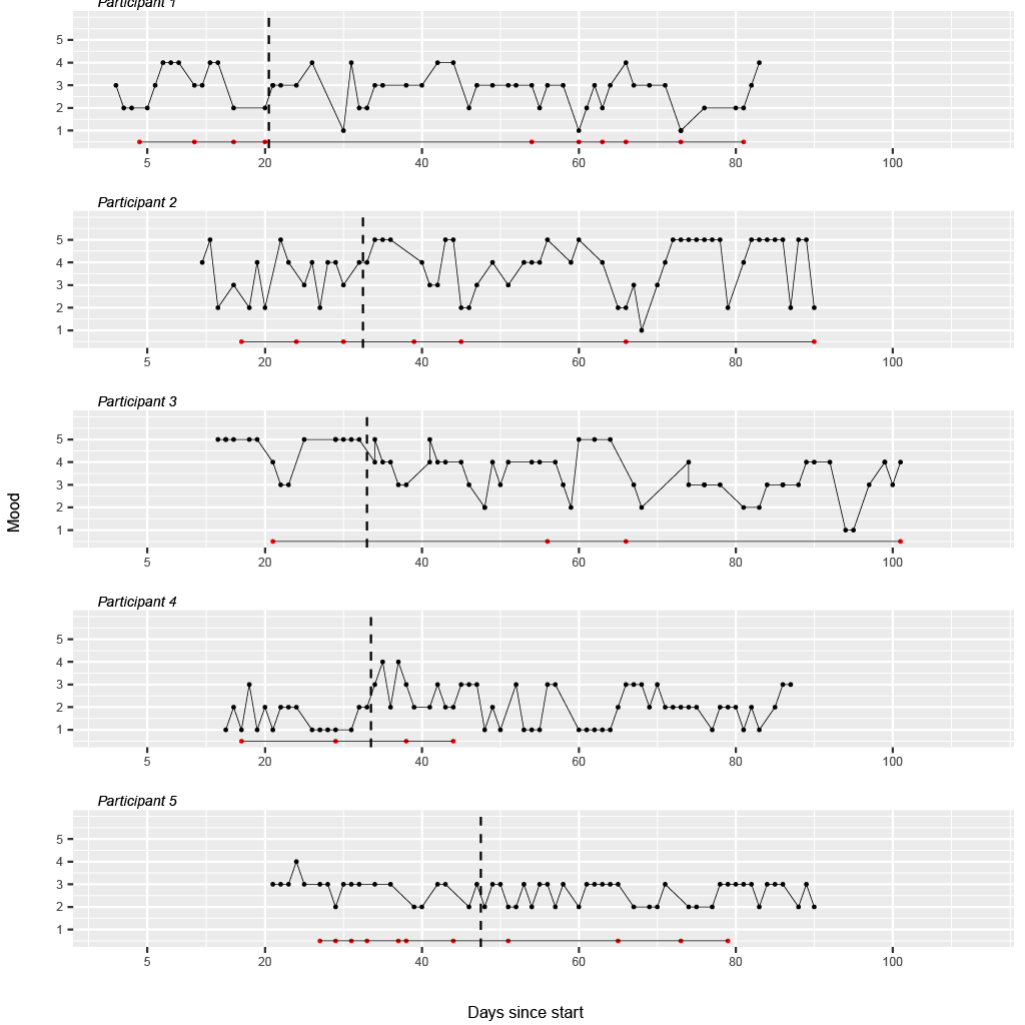
Daily reported mood scores for participants 1 to 5 in the Budd app effectiveness study.

**Figure 3:**
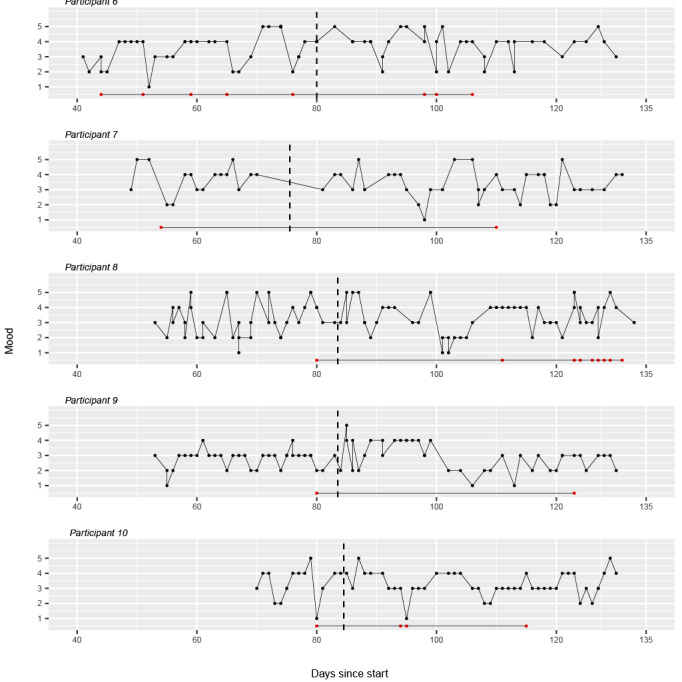
Daily reported mood scores for participants 6 to 10 in the Budd app effectiveness study.

Figures 2 and 3 illustrate the daily mood scores for each of the 10 participants. Each plot represents the longitudinal data of an individual participant, with the days of the study on the horizontal axis and the mood scores, scaled from 1 to 5, on the vertical axis. The dashed line indicates the transition from phase A (no intervention) to phase B (intervention). The red dots represent the reported chemsex sessions.

For participant 1, during both phase A and B of the study, many fluctuations in mood can be observed. The participant consistently reports a low mood score right before or after their participation in a chemsex session, except for the low score on day 30, which does not occur at the same time as a reported chemsex session.

For participant 2, substantial mood fluctuations are observed during both phases of the study. During phase A, mood scores are generally lower and show variability. With the start of the intervention phase B, mood scores appear to increase and demonstrate some stabilization in the higher range, although variability remains with some notable dips. In phase A, attendance at chemsex events was followed by subsequent low mood scores. However, in phase B, this was no longer observed, except for the lowest score on day 66. Overall, it can be observed that mood states improved in phase B compared with phase A.

For participant 3, as phase B progressed, there was a noticeable decline in mood state compared with phase A. The dip in mood state during phase A occurs at the same time as the participant’s participation in a chemsex event on day 21.

For participant 4, during phase A of the study, participant 4 consistently reported low mood scores. In the early part of phase B, there is an upward trend with some peaks suggesting an improvement, but this is followed by marked variability and several low points, indicating continued instability in his mood state. Notably, the lowest scores were not related to the moments when the participant engaged in chemsex activities.

Participant 5 reports generally low mood scores throughout the study. Visually, there is a slight decrease from phase A to phase B. The state remains fairly stable throughout the study.

For participant 6, the mood tracking for participant 6 shows fluctuations throughout both phases A and B. The mood scores vary widely, indicating unstable mood patterns. There is a general upward trend in the mood scores during phase A.

For participant 7, in phase A, there is a variation in mood, with considerable fluctuations indicating a period of instability. The variability in mood persists into phase B.

Participant 8 exhibits a varied mood pattern. With the start of phase B, an initial increase in mood scores is visible. However, toward the end of phase B, there’s noticeable variability with mood scores dipping and rising sharply.

For participant 9, the mood tracking shows a high degree of variability in phase A. The overall trend in phase B shows a gradual leveling of mood, yet with continued variability.

For participant 10, Mood assessments of participant 10 show fluctuations in phase A. In phase B, while mood continues to fluctuate, there is a noticeable upward trend toward the end of the phase.

### Self-Reported Safe Dosing Behavior

**Figure 4:**
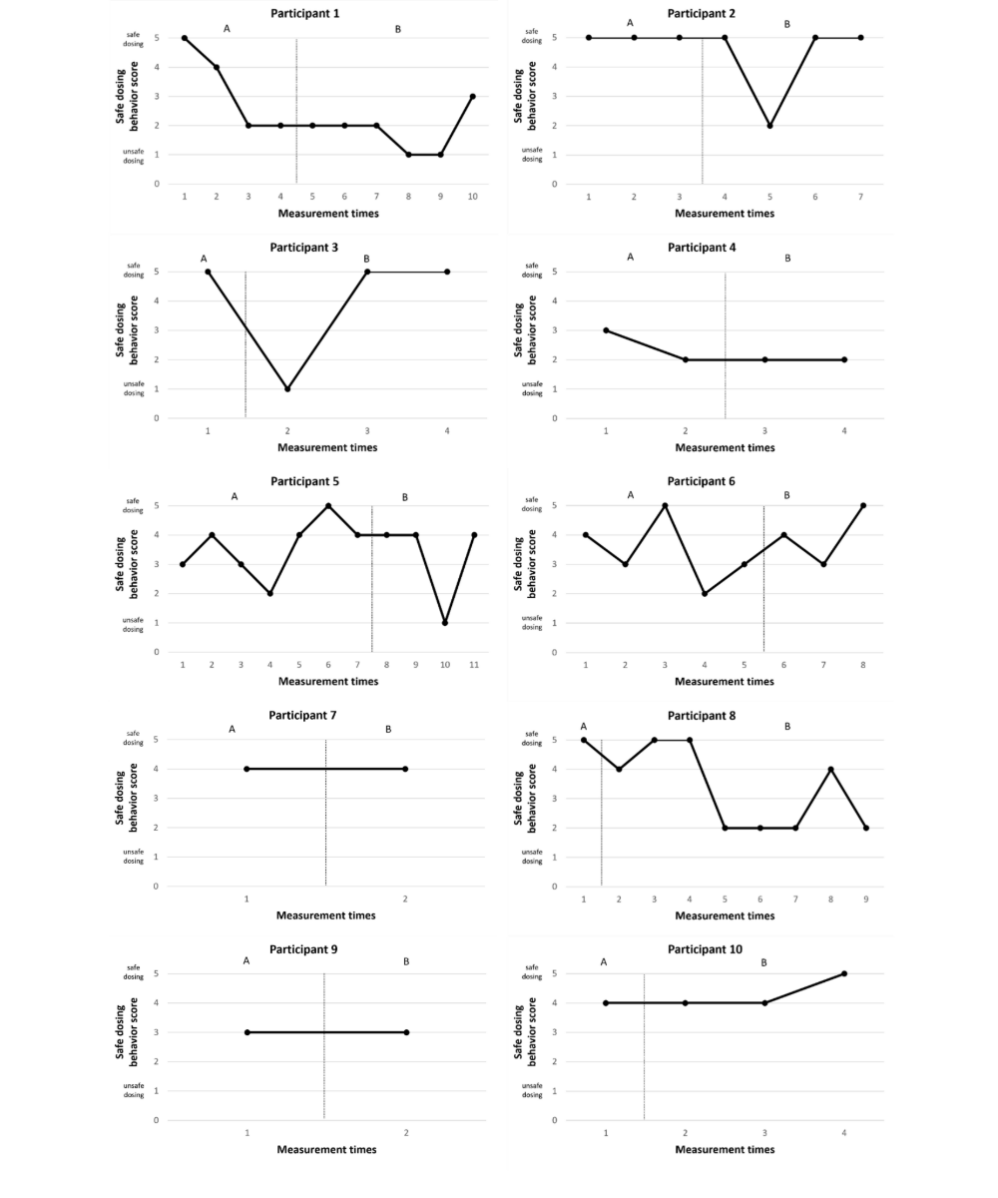
Safe dosing behavior scores for participants 1 to 10 in the Budd app effectiveness study.

Figure 4 displays the self-reported safe dosing behavior scores of the participants. The dashed line indicates the transition from phase A (no intervention) to phase B (intervention).

Participants 5, 6, and 8 show variable patterns in their safe dosing behavior throughout the study. Participants 2 and 3 initially showed a decrease in safe dosing scores following the start of the intervention, which later returned to maximum levels. Participant 4 consistently reported low safe dosing scores, maintaining this level throughout both the baseline and intervention phases. Participants 7, 9, and 10, who provided few data points, demonstrated consistent safe dosing scores during both phases. Notably, participant 2’s safe dosing behavior declined initially but improved in the last recorded chemsex session.

### App Use

Table 3 provides an overview of the app use data per participant.

**Table 3:** Use data for the Budd app among participants in the effectiveness study.

Participant number	Number of sessions	Pages viewed per session	Average session duration	Total duration	Total pages viewed
1	60	4.78	0 min 59 s	0 h 59 min	287
2	71	5.28	1 min 46 s	2 h 06 min	375
3	81	7.52	2 min 29 s	3 h 21 min	609
4	69	4.94	1 min 4 s	1 h 14 min	341
5	50	4.12	58 s	0 h 48 min	206
6	66	6.79	2 min 5 s	2 h 17 min	448
7	59	7.68	2 min 32 s	2 h 29 min	453
8	51	5.53	1 min 13 s	1 h 02 min	282
9	97	5.71	1 min 58 s	3 h 49 min	554
10	82	6.12	2 min 41 s	3 h 42 min	502

### Behavioral Determinants

#### Overview

Table 4 provides an overview of the change in behavioral determinants measured at baseline and at the end of the study.

**Table 4:** Behavioral determinants measured at baseline (pre) and at the end of the study (post) for participants in the Budd app effectiveness study.

Participant	Behavioral intention			Awareness of risk behavior			Self-efficacy	
	Pre	Post	Pre		Post	Pre		Post

1	4.25	3.75 (–11.76%)	5		3.33 (–33.4%)	4		3.5 (–12.5%)
2	3.75	4.75 (+26.67%)	3.33		4.67 (+40.24%)	4.25		4.5 (+5.88%)
3	4.5	5 (+11.11%)	4		4 (0%)	4		4.25 (+6.25%)
4	3.25	3.75 (+15.38%)	3.33		2.6 (–21.92%)	2.75		2.75 (0%)
5	4.25	4.5 (+5.88%)	4		3.67 (−8.25%)	4.25		3.25 (–23.53%)
6	4.5	4.25 (–5.56%)	4		4.3 (+7.5%)	3.25		3.5 (+7.69%)
7	3.75	4.25 (+13.33%)	3		4 (+33.33%)	4		3.5 (–12.5%)
8	4.25	4.25 (0%)	4.33		4.67 (+7.85%)	3.5		4 (+14.29%)
9	3.25	4.25 (30.77%)	3.67		3.33 (–9.26%)	3		3.75 (+25%)
10	4.75	4.75 (0%)	4.67		4 (–14.35%)	4.5		4.25 (–5.56%)

#### Behavioral Intention

Preintervention, the participants generally reported high levels of intention to engage in safer drug behaviors, with a mean score of 4.05 (SD 0.52) across participants. For instance, participant 1 initially reported a score of 4.25, indicating a strong initial intention to engage in safer behaviors.

Postintervention, after using the Budd app, there were observable changes in participants’ behavioral intentions. Of 10 participants, 6 showed an improvement in their behavioral intention scores. Participants 8 and 10 maintained their scores, which were already high preintervention with scores of 4.25 and 4.75, respectively. Participant 1 and participant 6 showed a slight reduction.

#### Awareness of Risk Behavior

Before the intervention, participants generally exhibited a relatively high level of awareness of their risk behavior regarding drug harm reduction practices, with an average score of 3.93 (SD 0.63) across all participants.

Notably, half of the participants reported a decrease in their awareness of risk behavior after the intervention. For example, participant 1 reported a decrease from 5 preintervention to 3.33 postintervention, and participant 4’s score dropped from 3.33 preintervention to 2.6 postintervention.

#### Self-Efficacy

Before the intervention, participants exhibited varying levels of self-efficacy, with an average preintervention score of 3.75 (SD 0.59) across all participants.

After the intervention, participant 2’s score increased from 4.25 preintervention to 4.5 postintervention, and participant 9’s score improved from 3 to 3.75.

However, a number of participants reported a decrease in their self-efficacy scores after using the Budd app. For example, participant 1’s score decreased from 4 preintervention to 3.5 postintervention, and participant 5's score reduced from 4.25 to 3.25.

#### Knowledge

Table 5 presents the pre- and post-scores, absolute improvement in scores, and the percentage improvement relative to their prescores of each of the 10 participants.

**Table 5:** Knowledge scores at baseline (pre) and at the end of the study (post) for participants in the Budd app effectiveness study.

Participant	Prescore (out of 34)	Postscore (out of 34)	Score improvement	Percentage improvement
1	18	19	1	5.56
2	17	20	3	17.65
3	17	21	4	23.53
4	20	26	6	30.00
5	26	30	4	15.38
6	25	28	3	12.00
7	21	28	7	33.33
8	28	29	1	3.57
9	17	25	8	47.06
10	28	33	5	17.86

All participants showed a positive improvement in their drug harm reduction knowledge after using the Budd app. The mean percentage improvement across all participants was 20.59% (SD 13.30%). While participant 9 exhibited the greatest-percentage improvement at 47.06%, it is important to note the context for each participant’s progress. For instance, although participant 8 had the smallest percentage improvement at 3.57%, his initial score was already high (28/34), leaving little room for improvements. By contrast, participants with a lower initial score, such as participant 9, had more potential to show considerable percentage gains.

## Discussion

### Principal Findings

This study evaluated the effectiveness of the Budd app in promoting harm reduction measures related to substance use among gbMSM participating in chemsex. The study combined a single case and pre-post experimental design. Initial findings suggest an impact on participants’ knowledge of chemsex substances. This increase in knowledge is a positive indicator of the app’s educational impact.

However, outcomes in behavioral intention, self-efficacy, and awareness of risk behavior are less uniform. This variability was further highlighted regarding safe dosing practices, indicating a complex relationship between these determinants and actual behavioral change. Participants may be sensitized and aware of the complex interplay between substances and how it challenges their situation. This may imply that they estimate their ability to cope or adapt their behavior less optimistically after having been better informed. While some participants showed a shift toward safer practices, others displayed variability or no change. This diverse range of responses underscores the individualized nature of behavior change in the context of chemsex.

Participant engagement with the app also varied, suggesting that the level of interaction may influence the intervention’s effectiveness. Moreover, mood fluctuations reported by participants could reflect broader psychosocial factors impacting both app engagement and behavior change.

### Comparison With Prior Work

#### Nuanced Behavioral Outcomes

In light of the growing role of mHealth interventions in public health, our study reveals valuable insights into their effectiveness within the specific context of chemsex among gbMSM. Our results contrast with the study by Choi et al [31], which demonstrated a strong behavior change among gbMSM following a brief web-based harm reduction intervention. While they report a reduction in both intention and actual chemsex behavior, our study shows a more nuanced picture. Our findings highlight the tailored nature of behavior change interventions for chemsex.

#### Knowledge

Improved knowledge was a consistent finding in our study. We observed a consistent improvement in knowledge among participants, with a mean increase of one-fifth compared with baseline value. These results align with existing literature on mHealth interventions, demonstrating that these tools can be effective in improving health-related knowledge [57-59].

A key focus of the Budd app lies in providing reliable information in a nonjudgmental way. The app includes several practical applications designed to enhance knowledge and promote safer practices. Regarding drug use, these include information on commonly used chemsex drugs, a drug combination tool, articles on drug harm reduction, and what to do in case of an emergency. These components specifically address the gap in reliable and accessible information about chemsex, thereby contributing to the observed increase in knowledge among our study participants. Crucially, all this is done in a manner that avoids judgment, criminalization, or repression of chemsex, fully respecting the autonomy of the users.

The gap in access to reliable information is reflected in the “chemsex circle of experts and novices,” a phenomenon in which knowledge about chemsex and harm reduction is often disseminated within the community itself based on personal experiences rather than being acquired from health care providers [60]. Research points to a consistent trend that gbMSM and lesbian, gay, bisexual, transgender, and queer+ individuals predominantly turn to informal networks and web-based sources for harm reduction information. In Australia, where a group of self-identified sexual minority men revealed that many predominantly consult friends, casual partners, strangers on hookup apps, and web-based communities for health information [23]. Among a group of Swedish gbMSM, knowledge of drug harm reduction strategies is mainly shared within the community and on the web instead of through health care providers [61]. A systematic review also shows that the internet is the primary source of health information for lesbian, gay, bisexual, transgender, and queer+ individuals, who also rely on schools, peers, and parents [62].

The search for informal sources of information is guided by chemsex users’ fear of being judged, and their subsequent preference for more anonymous or informal sources of expert advice [23,32]. This reliance on community-based knowledge transfer, which is often perceived as less reliable despite their widespread use [23], may prevent newly self-identified or isolated gbMSM from getting access to reliable knowledge needed for informed choices about safer chemsex practices. This need for new, reliable, and trustworthy sources of chemsex harm reduction information is emphasized in other studies [63,64] and during our in-depth interviews [32]. The improvement in knowledge scores postintervention suggests that the Budd app is effectively fulfilling a critical need for reliable and accessible information within the gbMSM community engaged in chemsex.

#### Behavioral Intention

Next to providing information, the Budd app focuses on “goal setting” and “goal appraisal” [65]. These behavior-change methods are directed toward influencing the users’ behavioral intentions regarding harm reduction practices when using drugs. By enabling users to set personal harm reduction goals within the app, we provided a structure for self-directed change.

In our study, we observed a variable impact of the Budd app on participants’ behavioral intentions regarding harm reduction during chemsex sessions. On average, participants reported high baseline intention scores for safe drug use (mean 4.1). Postintervention, some participants showed improvements and some slight decreases in their intentions.

While other studies often show a positive and significant correlation between behavioral intentions and actual behavior [66,67], our findings were more ambiguous. The high intention scores in our study did not consistently translate into behavior change, such as safe dosing behavior. A possible explanation is that when being under the influence of drugs, behavior is perceived less impactful, blurred by intoxication, and thus, less mediated by intentions.

According to the study by Ouellette and Wood [66], the complexity and variability of contexts make conscious decision-making more challenging. Context plays a pivotal role in chemsex situations. Factors such as the physical and social environment, peer pressure, intoxication, and the allure of immediate gratification from high-risk behaviors can predispose individuals toward automatic, rather than intentional, actions. These influences can overshadow both the knowledge and intentions of harm reduction, resulting in a gap between what individuals know and what they actually practice in the context of chemsex [32,46].

In health behavior, people convert their “good” intentions into action in only 53% of the time [68]. This discrepancy between intentions and actual behavior change is referred to as the intention-behavior gap, reflecting that forming goal intentions is necessary yet insufficient for goal attainment [69,70]. When considering chemsex, the effect of this gap may even increase, as reported in the study by Hibbert et al [71] when they conclude that engagement in chemsex can lead to actions that participants would not consider when sober.

#### Bridging the Gap

The app was built consistent with the self-control framework as presented elsewhere [65]. This framework emphasizes that chemsex users reduce the chemsex associated harms by planning (“before”), act upon their plan (“during”), and reflect (“after”). This is translated into specific features in the app, including a planning tool (“goal setting”), a specific design of the app during a session (“goal enactment”), and a reflection of their planned behavior (“goal appraisal”). “Goal adjustment,” the final step in the self-control process, is achieved via the planning of the next chemsex session [65].

While the Budd app addresses individual behavioral factors, it emphasizes less on the broader contextual influences. mHealth interventions such as ours, targeting HIV and STIs, sexual risk, substance use, and mental health among gbMSM, are typically guided by theories such as the information-motivation-behavioral skills model and social cognitive theory. Although both theories acknowledge the importance of this wider context, it is less integrated in our Budd app [72].

Our study’s focus on individual factors prevented a full exploration of these broader social determinants. The gap in addressing these factors in the app’s design might explain the limited impact observed in behavioral change. In addition, the limited time that people used the app may have led to a reduced effect. Future developments of the Budd app could benefit from incorporating these wider contextual elements, enhancing its effectiveness in managing the complex health behaviors among gbMSM engaged in chemsex.

#### User Engagement

The most commonly collected measures of engagement in eHealth and mHealth interventions are system use data [73]. If users do not interact with the interventions features, exposure to behavior change intervention components will be limited and less likely to influence the behavioral determinants that lead to health behavior engagement [74]. Participants 1 and 5 used the Budd app the least; however, they showed the highest levels of engagement in chemsex sessions. This seems to indicate a dose-response relationship between app use and the effectiveness of the intervention. Particularly in the case of participant 1, we see high engagement in risky behavior and a regression across all behavioral determinants, except for a negligible gain in knowledge, which further underscores the lack of the intervention’s effectiveness.

Participant 5 also showed a decline in awareness of risk behavior and self-efficacy. However, he maintained a generally high level of dosing safety and scored high on the knowledge quizzes, indicating that preexisting safer behaviors were preserved. Despite low app use, the slight improvement in intention, which was already high, suggests that the app may have reinforced existing positive behaviors rather than initiating changes.

These outcomes across both participants illustrate a complex dose-response relationship where low use of the intervention does not necessarily mean a deterioration in risk behaviors. This pattern challenges the typical expectation that increased engagement with a health intervention directly correlates with better outcomes. Instead, it highlights the nuanced ways in which individual characteristics and preexisting behaviors interact with intervention strategies, affecting their overall impact. We recognize that system use data, while commonly used, provides a limited view of user engagement [75].

We note that aspects of engagement other than use data are important. These quantitative measures capture the extent of interaction but not the quality of engagement. This approach overlooks the users themselves and their circumstances [76]. Engagement levels can be affected by the way content is structured and presented, environmental pressures, symptom burden, etc [77]. This broader context is essential to understand why users may or may not stay engaged with an app. Therefore, integrating qualitative assessments such as user satisfaction surveys and interviews can enrich our understanding by revealing how the app meets user expectations and its usability in real-world settings. In future studies, we plan to integrate these assessments to provide a more comprehensive understanding of engagement.

### Strengths and Limitations

Our study offers several strengths, including the innovative use of an mHealth intervention tailored to address the unique needs of gbMSM engaging in chemsex. The use of a combined approach using a pre-post and SCED, we gained insights into individual behavioral changes. This is particularly valuable given the complex nature of chemsex behavior. SCEDs are well-suited for examining complex behavior and individual differences [39], making them especially relevant in the context of chemsex research [7]. In addition, these designs enable the study of real-world contexts, which is critical when examining chemsex behavior due to its occurrence in private and stigmatized communities [8]. By focusing on a group of 10 participants, our study offers insights into the nuanced differences in chemsex behavior and the effects of our intervention on each individual. By integrating visual and statistical findings, we sought to provide a comprehensive overview of the intervention’s impact on gbMSM’s harm reduction practices during chemsex.

However, some limitations need to be considered. It is important to acknowledge that the frequency of data collection in the SCED was low, which could impact the study’s internal validity and reliability. In addition, the pre-post study component was limited by the number of participants. The 6-week study period, while providing initial insights, might not be sufficient to evaluate long-term effects of the intervention. These factors underline the preliminary nature of the findings and underscore the necessity for future research. Subsequent studies will focus on increasing the study period and the number of participants. This approach is expected to provide stronger evidence of the intervention effects and enhance the overall validity of the findings. Furthermore, reliance on self-reported data introduces biases. Social desirability bias is particularly significant, as participants might alter their responses due to societal norms and stigmas associated with chemsex, leading to systematic reporting errors. Recall bias also affects reliability, as participants may struggle to accurately recall and report behaviors from chemsex sessions. In addition, the tendency to overreport or underreport behaviors further skews the data. These issues underscore the complexities of interpreting self-reported data in this sensitive context.

In light of these results and existing literature, the role of digital interventions in promoting harm reduction behavior must be critically examined. Further research, preferably with a larger sample size and a longitudinal approach, is necessary to understand and optimize the effects of such interventions for harm reduction in the context of chemsex.

### Conclusions

In conclusion, while the Budd app demonstrated potential benefits in increasing knowledge and influencing some behavioral determinants, the findings should be viewed as preliminary. The limitations in study design underscore the need for further research. Subsequent studies should increase the number of participants and the frequency and robustness of data collection to extend these findings. Our research contributes to the growing body of knowledge on digital interventions in public health, particularly in the realm of sexual health and harm reduction among gbMSM. The findings emphasize the potential of mHealth interventions in addressing complex health issues, while also highlighting the challenges in achieving consistent behavioral change through digital platforms alone.

## Appendix

Multimedia Appendix 1 Additional tables.

Multimedia Appendix 2 Chemsex knowledge questionnaire.

Multimedia Appendix 3 Questions on behavioral determinants.

Multimedia Appendix 4 Demographic and risk behavior questionnaire.

## Abbreviations

gbMSM: gay, bisexual, and other men who have sex with men
IMP: intervention mapping protocol
ITM: Institute of Tropical Medicine
mHealth: mobile health
PrEP: preexposure prophylaxis
SCED: single-case experimental design
STI: sexually transmitted infection

## Data Availability

Data on mood and safe-dosing behavior analyzed during this study are included in this published article. Data on behavioral determinants and app use are available from the corresponding author on reasonable request.
