# Supplementary material for: Assessing the Effectiveness of an mHealth Intervention to Support Men Who Have Sex With Men Engaging in Chemsex (Budd): Single-Case and Pre-Post Experimental Design Study
Source: JMIR Form Res. 2024 Oct 4;8:e56606. doi: 10.2196/56606 (PMC11489797; doi:10.2196/56606)
Supplement: Multimedia Appendix 1 [file formative_v8i1e56606_app1.pdf]

Multimedia Appendix 1: Supplementary overview tables

Duration of baseline and intervention phases

Table 1: Duration of baseline and intervention phases for each participant

Participant	Start date phase A	Number of days phase A	Start date phase B	Number of days phase B
1	15 October 2021	19	3 November 2021	63
2	26 October 2021	20	15 November 2021	59
3	28 October 2021	18	15 November 2021	64
4	29 October 2021	19	17 November 2021	72
5	4 November 2021	27	1 December 2021	42
6	23 November 2021	44	6 January 2022	46
7	1 December 2021	33	3 January 2022	51
8	6 December 2021	31	6 January 2022	47
9	6 December 2021	31	6 January 2022	47
10	23 December 2021	15	22 February 2022	46

Mean mood scores

Table 2: Mean mood scores of participants of the Budd effectiveness study during Phase A (baseline) and Phase B (intervention)

Participant	Mean mood score phase A	Mean mood score phase B
1	3	2.72
2	3.44	3.96
3	4.67	3.40
4	1.53	2.13
5	2.8	2.6
6	3.68	3.77
7	3.69	3.36
8	3.24	3.39
9	2.76	2.90
10	3.36	3.28
