# Supplementary material for: Assessing the Effectiveness of an mHealth Intervention to Support Men Who Have Sex With Men Engaging in Chemsex (Budd): Single-Case and Pre-Post Experimental Design Study
Source: JMIR Form Res. 2024 Oct 4;8:e56606. doi: 10.2196/56606 (PMC11489797; doi:10.2196/56606)
Supplement: Multimedia Appendix 2 [file formative_v8i1e56606_app2.pdf]

Multimedia Appendix 2: Knowledge questionnaire

1. GHB/GBL is:

- A depressant
- A stimulant
- A hallucinogen
- An analgesic

2. What is GHB?

- A substance found naturally as a neurotransmitter in our bodies.
- A common solvent in chemistry that is used as a stain remover, wheel cleaner, paint stripper, …
- Nature product stemming from South America
- Liquid, easily dosed, chemically derived.

3. GBL overdose:

- Is very rare because someone would have to drink a lot of it
- Causes racing heart rate, hallucinations, fever, and stroke
- Causes vomiting, headache, unconsciousness, and fatal respiratory arrest
- Causes some minor nervousness, sweating and nausea

4. How long after ingestion does GHB usually begin to work?

- Immediately
- 5 - 30 minutes
- 30 - 60 minutes
- 1 – 2 hours

5. How quickly does Ketamine cause loss of control and/or unconsciousness?

- Less than one minute
- Five minute
- Fifteen minutes
- Half an hour

6. Which of the following is not true about Ketamine?

- It causes a splitting from reality
- It effects are highly dose-dependent
- It has no legitimate medical use
- It causes hallucinations

7. One of the dangers of using ketamine recreationally is…

- Increased heart rate
- Decreased heart rate
- Increased respiratory rate
- Decreased respiratory rate

8. The main acute risks when using XTC are:

- Anxiety and high body temperature
- High body temperature and racing heart rate
- High body temperature and dehydration
- Racing heart rate and dehydration

9. If you use XTC it is recommended to drink 1 glass per hour, what is best to drink?

- Beer
- Water
- Energy drink
- Doesn't really matter, as long as you drink

10. What diseases can you get by sniffing through someone else's snorting tube?

- Chlamydia
- Rubella
- Hepatitis B or C
- No diseases, the cocaine disinfects the tube during use

11. How long after consumption does cocaine usually begin to work?

- 15 - 45 seconds
- 1 – 5 minutes
- 5 – 15 minutes
- More than 15 minutes

12. How long do the effects of snorting cocaine last?

- Approximately 10 minutes
- Approximately 30 to 45 minutes
- Approximately 1 hour
- Approximately 2 hours

13. Which of the following is not a short-term effect of cocaine use?

- Appetite suppression
- Increased libido
- Hair loss
- Higher blood pressure and faster heartbeat

14. What is true about the combination of cocaine and sex?

- A large quantity of coke makes an orgasm more intense
- It only gives the energy to go on longer, nothing else
- With coke it is easier to get/keep an erection
- In small doses it has an enhancing effect on lust, but a reducing effect on performance

15. Amphetamines are:

- Psychomotor depressants
- Psychomotor stimulants
- Hallucinogens
- Cannabinoids

16. Which of the following is caused by long-term amphetamine abuse?

- Memory loss
- Liver damage
- Delusions and paranoia
- All of these

17. What is not an effect of speed?

- Less desire for sex
- Depressive feelings
- Greater appetite
- Psychosis

18. Methamphetamine is a:

- Psychomotor depressant
- Psychomotor stimulant
- Hallucinogen
- Cannabinoid

19. A methamphetamine experience lasts about how long after the initial rush?

- Two or three hours
- Two to six hours
- Two to twelve hours
- Two to eighteen hours

20. Which of the following does short-term methamphetamine not cause?

- Appetite suppression
- Increased libido

- Heavy sleep with bizarre dreams
- Focus enhancement

21. What is the link between cannabis use and psychosis?

- Cannabis use may trigger psychosis
- Cannabis can only trigger a short-lived psychosis
- There is no association between cannabis use and psychosis
- Cannabis use may cause a psychosis in healthy individuals

22. Cannabis gives a pleasant feeling because:

- It directly stimulates the reward centre in your brain.
- Cannabis affects your memory, causing you to forget negative things.
- The reward centre in your brain is stimulated indirectly.
- Cannabis influences your perception, causing you to look at everything in a positive way.

23. Which statement about cannabis is correct?

- Cannabis affects your short-term memory and ability to concentrate, this will recover after quitting.
- Cannabis permanently affects your short-term memory and ability to concentrate.
- Cannabis affects your long-term memory.
- Cannabis has no effect on your memory.

24. If you combine speed with alcohol…

- you feel less drunk.
- nothing happens, these drugs do not influence each other's effects.
- you will feel less exhilarated.
- you feel less of the speed and the alcohol.

25. What does it mean when two substances are synergistic?

- The combination of these substances leads to a larger effect than expected.
- The combination of these substances leads to a smaller effect than expected.
- The combination doesn't have an effect on both substances
- The combination cancels out both effects, so it feels like you're sober

26. The combination of cocaine and alcohol leads to:

- Less risk, cocaine partially cancels out the effect of alcohol
- As much risk as taking them separately
- No risk, the effects cancel each other out
- More risk, the combination forms a substance that is more harmful and addictive

27. Combination alcohol and cocaine:

- This is a low risk combination.
- This combination can lead to excessive drinking (risk of liver damage and increased dehydration)
- This combination has a high risk of causing nausea and vomiting.
- This combination leads to extreme and severe health risks

28. What is true about the combination of speed and alcohol?

- Speed counteracts the narcotic effects of alcohol
- Alcohol and speed do not influence each other's effects
- Both effects cancel each other out
- Alcohol enhances the effect of speed

29. What is not true about the combination of speed and GHB?

- The use of speed and GHB is extra harmful for the body
- The effects of GHB weaken the effects of speed.
- GHB does not give the user a hangover the next day
- The risk of passing out is increased.

30. Combination GHB/GBL and MDMA/XTC:

- This is a low risk combination.
- Both effects reinforce each other; an increased risk of overdose
- This combination can strongly disinhibit you sexually. You may start doing things you would not otherwise do
- This combination can lead to the suppression of breathing and unconsciousness.

31. What are the symptoms of water poisoning (multiple answers)?

A. Nausea
B. Dizziness
C. Sweating
D. Muscle cramps
E. Rapid heartbeat
F. Dry mouth

- Answer A, B and C are correct
- Answer A, B and F are correct
- Answer A, B and D are correct
- Answer A, B, E and F are correct

32. What should you do if someone becomes unconscious due to GHB?

A. Nothing, this is common and does no harm

B. Trying to wake up/keep the person awake
C. Lay the person on his/her side
D. Make the person vomit immediately
E. Monitor breathing
F. Call 112.

- Answer C,D and F are correct
- Answer B,C,E and F are correct
- Answer B to F are correct
- All answers are correct

33. What is the best thing to do if you have a hangover from the drugs you have taken?

- Drink a lot of coffee
- Taking anti-depressants
- Take extra vitamins, eat well and relax
- Nothing helps, you just have to sit out the hangover

34. What is the best meal to eat before leaving for a chemsex party?

- It is best not to eat anything in the hours before you leave.
- A light meal without carbohydrates.
- A hearty meal with carbohydrates, fats and proteins.
- A meal high in sugar and caffeine, like energy drinks and candy bars.
