# Supplementary material for: Assessing the Effectiveness of an mHealth Intervention to Support Men Who Have Sex With Men Engaging in Chemsex (Budd): Single-Case and Pre-Post Experimental Design Study
Source: JMIR Form Res. 2024 Oct 4;8:e56606. doi: 10.2196/56606 (PMC11489797; doi:10.2196/56606)
Supplement: Multimedia Appendix 3 [file formative_v8i1e56606_app3.pdf]

Multimedia Appendix 3: Behavioral determinants questionnaire

For each statement, participants can choose from 1 to 5, where 1 means ‘strongly disagree’ and 5 means ‘strongly agree’.

Behavioral goals	Behavioral intention	Self-efficacy	Awareness of risk behavior
Avoiding dangerous drug combinations	I intend to avoid combining drugs that can lead to dangerous interactions during chemsex sessions.	I am confident in my ability to avoid dangerous drug combinations during chemsex sessions.	I am aware of which kind of chems I am taking during a chemsex session.
Correct drug dosing	I intend to dose correctly during a chemsex sessions (measure and adhere to my personal drug dosage).	I am confident in my ability to dose drugs correctly during chemsex sessions (measure and adhere to my personal drug dosage).	I am aware of the specific quantities (in milligrams or appropriate units) of chems I take during a chemsex session.
Waiting long enough before taking a new dose	I intend to wait an appropriate amount of time before taking a new dose of any drug during chemsex sessions.	I am confident in my ability to wait for the right amount of time before taking a new dose of drugs during chemsex sessions.	I am aware of exactly at what times I take chems during the chemsex session.

Bringing essential items for drug harm reduction	I intend to bring essential items necessary for applying drug harm reduction measures to chemsex sessions	I am confident in my ability to bring essential harm reduction items for applying drug harm reduction measures to chemsex sessions.	/
