# Supplementary material for: Assessing the Effectiveness of an mHealth Intervention to Support Men Who Have Sex With Men Engaging in Chemsex (Budd): Single-Case and Pre-Post Experimental Design Study
Source: JMIR Form Res. 2024 Oct 4;8:e56606. doi: 10.2196/56606 (PMC11489797; doi:10.2196/56606)
Supplement: Multimedia Appendix 4 [file formative_v8i1e56606_app4.pdf]

Multimedia Appendix 4: Demographic and risk behavior questionnaire

Demographic information

1) To which of the following age categories do you belong?

☐ 18-24 years
☐ 25-29 years
☐ 30-39 years
☐ 40-49 years
☐ 50-59 years
☐ older than 60 years

2) What is your highest level of education?

☐ No secondary school diploma
☐ Secondary school diploma
☐ Graduate degree
☐ Professional bachelor
☐ Academic bachelor
☐ Master
☐ PhD

3) What is your current employment status?
☐ Unemployed
☐ Student
☐ Part-time employed
☐ Full-time employed
☐ Retired

Experience using mobile phone and applications

4) On average, how many hours a day do you spend on your mobile phone?
☐ Less than 30min
☐ 30min to 1h
☐ 1,5h to 2h
☐ 2,5h to 3h
☐ More than 3h

5) Do you use mobile health applications?
Examples of mobile health applications: Fabulous, MyFitnessPal, Lifesum, Headspace, Sleepcycle, Calm, etc.
☐ Yes
☐ No

6) How often do you use mobile health applications?
(if yes to the previous question)
☐ Rarely

☐ Once a week
☐ 2-3 times a week
☐ Once a day
☐ Several times a day

Participation in chemsex

7) Have you participated in chemsex in the past 2 months?
☐ Yes
☐ No

8) How often, on average, do you participate in chemsex?
☐ Daily
☐ More than once a week
☐ Weekly
☐ Monthly
☐ Less than once a month

9) Tick on the types of chems that you have taken while partying and playing:
☐ Alcohol
☐ GHB/GBL ('G')
☐ Cocaine ('Coke')
☐ Amphetamine ('Speed')
☐ Crystallized methamphetamine ('Tina', 'crystal meth')
☐ Mephedrone (4-MMC)
☐ 3-MMC
☐ Ecstasy/MDMA
☐ Ketamine
☐ Weed/Hash
☐ Poppers
☐ Other:
